# Tension between local, regional and national levels in Norway’s handling of COVID-19

**DOI:** 10.1177/14034948221075408

**Published:** 2022-02-03

**Authors:** Anette Fosse, Anders Svensson, Ingvill Konradsen, Birgit Abelsen

**Affiliations:** Norwegian Centre for Rural Medicine, Department of Community Medicine, UiT The Arctic University of Norway, Norway

**Keywords:** COVID-19, rural, chief medical officer, communicable disease control, crisis management, qualitative

## Abstract

**Aims:**

This study aimed to explore the tension between local, regional, and national authorities evoked by some rural municipalities’ decisions to impose local infection-control measures during the first weeks of the COVID-19 pandemic in Norway.

**Methods:**

Eight municipal Chief Medical Officers of Health (CMOs) participated in semi-structured interviews, and six crisis management teams participated in focus-group interviews. Data were analysed with systematic text condensation. Boin and Bynander’s interpretation of crisis management and coordination and Nesheim et al.’s framework for non-hierarchical coordination in the state sector inspired the analysis.

**Results:**

Uncertainty in the face of a pandemic with unknown damage potential, lack of infection-control equipment, patient transport challenges, vulnerable staff situation and planning of local COVID-19 beds were some of the reasons for rural municipalities imposing local infection-control measures the first weeks of the pandemic. Local CMOs’ engagement, visibility and knowledge contributed to trust and safety. Differences in perspectives between local, regional and national actors created tension. Existing roles and structures were adjusted, and new informal networks arose.

**Conclusions:**

**Strong municipal responsibility in Norway and the quite unique arrangement with local CMOs in every municipality with the legal right to decide temporary local infection-control measures seemed to facilitate a balance between top-down and bottom-up decision making. Tension between rural, regional and national actors that arose due to local infection-control measures, and the following dialogue and mutual adjustment of perspectives, led to a fruitful balance between national and local measures in Norway’s handling of the COVID-19 pandemic.**

## Introduction

The first case of COVID-19 in Norway was confirmed on 26 February 2020. National measures of intervention were announced on 12 March, often referred to as ‘closing down’ the country. Two weeks later, parliament passed a temporary law that gave the government extended authority to decide measures of intervention to reduce the consequences of the pandemic [1,2].

The first weeks of the pandemic can be described as a crisis in terms of the threat to core values, the safety of people or the functioning of critical infrastructures that must be urgently addressed under conditions of deep uncertainty [3].

Initially, the Norwegian government’s strategy was a strong national unified response. The incidence of infection at the beginning of the pandemic was geographically unevenly distributed. The capital region and other parts of Southern Norway were most afflicted. The national strategy was challenged when almost one third of the municipalities – mostly rural – chose to impose more strict local infection-control measures during the first two to three weeks of the pandemic, most of them reducing mobility by introducing quarantine rules for travellers from areas of Norway with infection outbreak [4]. The municipal Chief Medical Officers of Health (CMO) in charge of infection control were initiators, supported by the municipal crisis management (MCM) teams and the municipal council. In the wake of this, tension arose between local, regional and national authorities. National and regional authorities criticised the local measures, and the National Institute of Public Health recommended the municipalities not to take such actions [5]. On 29 March, the government advised the municipalities to revoke local measures due to the potential of inducing fear and uncertainty in the population [6]. Nevertheless, most of the municipalities decided to uphold the measures with minor legal adaptations. However, by mid-April, most of the municipalities considered local measures no longer necessary and did not renew them. Not long after, the Ministry of Health and Care Services stated that differentiation of national measures due to local situations could be necessary. Gradually, the Norwegian strategy changed to a balance between national and local measures based on continuous monitoring of the infection rate and a combination of advice and legislation. This approach has been successful. Norway has had among the lowest infection rates in the world and relatively low COVID-19-related mortality compared to other countries [7].

As researchers with a special interest for health services in rural areas, we share a concern regarding the balance between local and national levels. Furthermore, we share the preconception that medical and public-health competence embedded in the local community is a necessary supplement to national expertise.

### Aims

From this perspective, we set up a study to explore tensions between local, regional and national authorities evoked by the decisions in some rural municipalities to impose local infection-control measures during the first weeks of the COVID-19 pandemic in Norway. Our main focus was on the municipal CMOs’ role.

### Organisation of civil protection and communicable disease control in Norway

The Norwegian public administration has three levels: national, regional and local (Table I). The political system is characterised by strong sector ministries responsible for all activities and policy in their respective field, combined with extensive decentralised local governance. The national and regional levels have limited direct control over the municipalities [8,9].

**Table I.** Actors in Norwegian crisis management and communicable disease control.

*National level*	
National Government/Cabinet	Responsible for national crisis management and strategy
Ministry of Justice and Public Security	National responsibility for public security and safety
Ministry of Health and Care Services	Provides health and care for the population through legislation and budget allocations; responsible for secondary care and owner of hospitals
Ministry of Municipalities and Modernisation	Responsible for local government finances, rural and regional policy, local administration and so on
Institute of Public Health	Government agency and competence centre for public health and CDC; advisory function for CDC in the municipalities
Norwegian Directorate of Health	Executive agency, regulatory and implementing authority in areas of health policy, including CDC; advisory function for the government
Norwegian Directorate for Civil Protection	Coordinating agency and competence centre for civil protection; organised under Ministry of Justice and Public Security
*Regional level*	
State Governor	State’s representative in local counties; responsible for monitoring the decisions, objectives and guidelines set out by national authorities; includes the County Medical Officer (adviser on health issues)
Regional Health Authorities	Provide secondary health-care services and laboratory services through hospitals, owned by the Ministry of Health and Care Services
*Local level*	
Municipality	Provider of most welfare services, including primary health care, public health and infectious control; responsible for local CDC and crisis management
Chief Medical Officer of Health (CMO)	Medical doctor (often specialist in public health) who is an adviser for the municipality on public-health issues and also in charge of CDC

CDC: communicable disease control.

There are 356 municipalities in Norway. In 2021, nearly half of them had fewer than 5000 inhabitants, and 30% of the population lived in rural areas (centrality class 4–6; i.e. medium to least central), in many cases with a large distance to important service functions [10,11]. Norway has a strong and well-developed welfare state. The Regional Health Authorities owned by the Ministry of Health and Care Services deliver hospital care. The municipalities deliver most other basic welfare services, such as primary health care, kindergarten and primary education. There is a relatively high level of mutual trust among public sector organisations, and the population’s trust in government is high [12,13].

Crisis management in Norway is based on four steering principles: responsibility, equality, subsidiarity and collaboration (Table II) [14]. This, in combination with the decentralised local governance, is reflected in legislation and the organisation of public security and communicable disease control (CDC).

**Table II.** The four steering principles of crisis management in Norway.

Principle	Meaning
The principle of responsibility	The organisation responsible for a domain in a normal situation is also responsible for the necessary emergency preparedness and for handling extraordinary event
The principle of equality	The organisation operating during crises should be as similar as possible to the normal organisation
The principle of subsidiarity	Crises should be handled organisationally at the most basic (local) possible level
The principle of collaboration	Authorities, businesses or agencies have an independent responsibility for ensuring the best possible cooperation with relevant stakeholders

The Ministry of Justice and Public Security with its subordinate agency, the Directorate for Civil Protection and Emergency Planning, has superior responsibility for crisis management. The other ministries, regional health authorities and the municipalities have independent responsibilities for crisis management and public security in their areas of provision. The County Governor has an important role in crisis management as the state’s regional representative. Every municipality has a MCM team, which is activated in times of crisis.

The Act Relating to Communicable Disease Control (CDC Act) [3] gives municipalities extensive local responsibility and authority in preventing communicable diseases and handling outbreaks such as the COVID-19 pandemic [15]. The municipal councils in Norway have the authority to impose extensive and intrusive measures such as prohibiting assemblies, closing activities/businesses, isolation and so on. To our knowledge, most other comparable countries have a more centralised system. In CDC, the County Governor has a supervisory function but a limited operational role.

The Public Health Act and the CDC Act states that all municipalities must have one or several medical doctors as advisers for public-health issues and for infection control, so-called CMOs [15,16]. Especially in rural municipalities, these positions are often covered by the same doctor. The CMO has oversight over the prevalence of communicable diseases in the municipality and responsibility for handling outbreaks. The CMO has an advisory role but can decide and impose short-lived acute measures if necessary. This legal provision was the background for the local infection-control measures decided by rural municipalities during the first weeks of the pandemic.

Not all crises involve CDC, and not all CDC situations are crises, but COVID-19 triggered both action systems, resulting in some initial confusion as to whether the pandemic was to be seen as a health crisis led by the Ministry of Health and Care Services or a community crisis led by the Ministry of Justice and Public Security.

## Methods

### Study design and setting

During the first weeks after the COVID-19 pandemic reached Norway, several rural municipalities imposed local infection-control measures in addition to the national strategies. This action created tension between local, regional and national stakeholders in CDC and crisis management. We conducted a qualitative study based on individual interviews and focus-group interviews with local CMOs and MCM teams in May–August 2020.

### Participants

We recruited a purposive sample of eight CMOs and six MCM teams from a total of 10 rural municipalities using our network of rural CMOs and geographical knowledge. We aimed for geographical spread, variation in both local measures and experiences with COVID-19.

### Data collection

All interviews were performed online with Microsoft Teams. They lasted 30–90 minutes and were conducted according to established principles [17]. Based on interview guides, the moderator (A.F.) invited the participants to share experiences from the first five to six weeks after 12 March 2020. We asked for their experiences with national and local measures, and collaboration and communication locally, regionally and nationally. They were encouraged to elaborate on challenges and possible learnings points. The observer (B.A.) took field notes and posed additional questions. The interviews were audiotaped and transcribed verbatim.

### Analysis

Data analysis was performed stepwise, with new interviews supplementing the sample according to systematic text condensation [18]. Categories and findings were developed from the empirical data using editing analysis style [19]. Interpretation was inspired by Boin and Bynander’s perspectives of crisis management as ‘craftsman’s’ or ‘emergent’ [3], and by Nesheim et al.’s descriptions of four types of organisational distances [20]. All authors were involved in the analysis. We focused especially on the participants’ experiences regarding tensions between different stakeholders in CDC and crisis management.

### Ethics approval and consent to participate

Principles of the Declaration of Helsinki were followed. The Norwegian Social Science Data Services approved the study (# 904904). Informed consent was obtained from all participants.

## Results

Our data showed how insecurity arose facing a pandemic with unknown damage potential. Local CMOs and MCM teams acted to protect their local populations. Lack of infection-control equipment, patient transport challenges, vulnerable staff situations and arrangement of local COVID-19 beds were reasons for why some municipalities chose to impose local infection-control measures that were stricter than the national interventions. CMOs had a key role and created trust and safety through visibility and accessibility. Local and personal knowledge were important features. Tensions between public administrative levels led to both disagreement and collaboration. Established arenas and structures in the municipalities and between local, regional and national stakeholders were supplied with informal networks contributing to knowledge sharing and information. Our findings are elaborated below.

### Buying time with local infection-control measures

Many informants described how the first period of the pandemic was characterised by horror scenarios from abroad. This created uncertainty, insecurity and a feeling of lack of control at the local level. Informants described four main reasons for the local infection-control measures: need for time to establish necessary infrastructure and to provide enough staff; lack of infection-control equipment; experiences with bad winter weather, closed roads, long distances and difficult transport conditions if critically ill COVID-19 patients needed hospitalisation; and need for time to plan how COVID-19 patients should be cared for locally. Many felt that national authorities did not take these challenges seriously. One CMO commented on the unequal distribution of infection-control equipment and attention between hospitals and primary care:

I think that may have been the worst thing from the national point of view, the fact that they have not understood that there are rural areas in Norway . . . they have not understood this with local knowledge at all. And in addition, this huge focus on hospitals when everyone is to be treated in primary health care! (CMO 2)

Some of the CMOs said that they did not find it necessary to impose local infection-control measures, even though several of the neighbouring municipalities did. They emphasised that the national measures were sufficient, and that local quarantine decisions would lead to too great a restriction on people’s lives.

### Visible and steady CMOs created local trust and security

Many informants in the MCM teams expressed that the local CMOs created security both among the local decision makers and in the population by virtue of their competence and visibility. This was the case both in municipalities that decided upon local infection-control measures in and those that did not. Many emphasised the importance of the CMO being open and available to the population with information and having a short response time. Several CMOs wrote information letters on the municipality’s website or in the local press.

Many CMOs described the benefit of being able to combine professional knowledge about infection control with good knowledge of local conditions. Several MCM team members emphasised that the CMO had a natural place in the local crisis management, and that their medical expertise was necessary to create good and informed discussions. In many municipalities, members of the MCM teams had participated in local crisis management for many years and knew each other and local conditions well. They experienced it as a strength and a security to be a small municipality with short decision paths. All the CMOs described how they were listened to and that they were given a lot of authority and speaking time. Many described it as a new and inspiring experience that their competence and assessments were very important. Professional assessments weighed heavily in the local decisions on infection-control measures. One member of a MCM team said about the new role of the CMO:

I actually experienced that there was political will to stand firm in these matters and that they did it. . ., not only because it was politically interesting, but because it was based on infection control advice they received from the CMO. And she also briefed in all political meetings where decisions were to be made and gave status about the situation as she saw it and what recommendations to follow. So, I feel that it was both a political wish to take the important steps and dare to take the important steps at the same time as they should relieve some of the responsibility from the CMO so she would not stand alone in this situation. (MCM team member 3)

### Disagreement and cooperation between local, regional and national authorities

The municipalities experienced a lot of criticism from the national level for imposing local infection-control measures. There was much disagreement about their rationale and consequences. National authorities signalled that any local infection-control measures must not be too intrusive and questioned their proportionality. Many informants experienced that the national authorities did not acknowledge the differences throughout the country, and that they only viewed the country from the capital’s perspective. The fact that the Director of the Confederation of Norwegian Enterprise (NHO) announced nationally that he was annoyed with the local measures was commented on by several informants. It was perceived that national authorities threw doubt on the legality of the delegation authority, and this created uncertainty in many municipalities. At the same time, several CMOs described how they had meetings with local businesses and the local NHO and that the local measures were designed so that there was no conflict. Several informants considered that points of view and perspective had an impact on the national authorities’ reactions, as one interviewee put it:

Had it been the opposite, had it been Vesterålen or Tromsø that had been the epicentre, I am quite sure that Oslo would have been allowed to say that for a period we will encourage people from that area not to come to the city. But this was the other way round. (MCM team member 1)

Many of the informants had opinions about the role of the County Governor. Some had experienced good support when they needed to discuss local measures, while others felt that the County Governor had only been a disruptive element. Different County Governors emphasised different aspects in their contact with the municipalities. Some experienced that the County Governor in their county had more focus on control, while in other counties, guidance was the focus. There were also different opinions among our informants as to whether the County Governor should support the municipalities in their local decisions or be the state's spokesperson. Several called for the County Governor to contribute to coordination and cooperation. Many underscored that the situation was new to everyone, and that everyone had a steep learning curve. This is how a CMO described it:

It was not easy to be neither a CMO or a County Governor . . . Before you had time to make an announcement, you could risk that the rules were different. So, the durability of the advice was shorter than ever these times. (CMO 7)

### Roles and structures were adapted and established to share knowledge and support

The municipalities activated MCM teams in the beginning of March, many even before the national COVID-19 measures were launched. Our informants talked about frequent meetings and the establishment of smaller groups adapted to different needs. Many described an experience of a continuous state of emergency and almost 24/7 contact between the leaders responsible. A member of a MCM team explained how they established a new structure that made it easier for local businesses, sports teams and other associations to get in touch with the municipal department leaders to meet their need for information. One CMO described how they set up teams in the doctor’s surgery and regular meetings with the leaders for home-care services. She also described how she worked to establish stronger collaboration structures with the hospital based on the established schemes with strategic and professional collaboration committees. Another CMO experienced that the hospital often overruled the municipalities’ decisions:

We have had a lot of dialogue with the local hospital, I am also a member of the local professional cooperation body, and it’s a bit like, yes, we hear what you say, but, yes, this is how it will be. (CMO 3)

All mayors, heads of municipal affairs and CMOs were summoned fairly quickly to online meetings with the County Governor. Informants described these meetings as chaotic to begin with, but that they became more useful as the County Governor divided participators into smaller groups that corresponded with the municipal regions and local hospitals’ catchment areas. The meetings that arose between the Chief County Medical Officers, local health trusts and the corresponding municipals’ CMOs were described by several informants as useful because they provided an opportunity for discussions about reallocation of infection-control equipment, personnel and other mutual issues.

Informants from all levels talked about the formation of informal networks between professionals both internally in the municipality and with other municipalities. The communication took place in chat groups. MCM team informants told how they came together with the other municipalities in the region to clarify how they should handle local decisions about infection-control measures. In some regions, measures were similar, while in others, different choices were made. One informant described the collaboration as follows:

We worked very hard towards the common goal, which was to secure our citizens. And put in place good arrangements and solutions so that we could meet this which we did not know what was, which was very unknown to us and which we all feared. (MCM team member 3)

CMOs organised internal networks in their region and participated in national networks and chat groups. They described the chat arena as informal with a low threshold for exchanging information about decisions, routines and experiences, and airing issues that provided new knowledge, inspiration, correction and support.

## Discussion

Widespread uncertainty in the face of a pandemic with unknown damage potential, lack of infection-control equipment, patient transport challenges, vulnerable staff situation and planning of local COVID-19 beds were some of the reasons for rural municipalities imposing local infection-control measures during the first weeks of the pandemic. Local CMOs’ engagement, visibility and knowledge contributed to trust and safety in the study municipalities. Differences in perspectives between local, regional and national actors created tension. Existing roles and structures were manifested and adjusted, and new informal networks arose.

### Tension and balance

Lægreid and Rykkja describe how ‘local authorities are responsible for providing a broad range of services, and that the local democratic tradition and independence is strong in Norway. This implies that horizontal coordination within each municipality is strong, but that there are tensions between central state and local government’ [21]. This is clearly demonstrated in our study. The local infection-control measures that were introduced in the first weeks of the pandemic were rooted in a strong tradition in Norway of local democracy and authority, deeply founded in our Constitution, the Local Government Act, the CDC Act and the principles of crisis management. An interesting observation was how this legal local self-governance seemed to surprise and frustrate national authorities. Another observation was how the County Governor’s role and performance was experienced as either helpful or intrusive by our informants. Boin and Bynander describe how formal authorities may trigger processes of collaboration or fuel fragmentation when they arrive in a crisis situation to impose collaboration [3]. Some of our informants expressed frustration over national authorities’ seeming neglect of rural perspectives and challenges. This is in accordance with the description of geographical and cognitive distances in Nesheim et al.’s analytical framework [20], and also in tune with Fors’ concept of geographical narcissism [22]. The danger of these types of distances is that the ability to understand each other’s perspectives is weakened. Heterogeneous local practices can stand in the way of sensible cooperation and coordination but, on the other hand, national authorities’ lack of insight into local situations can lead to less suitable measures. An international study mapping participation in decision-making processes during the first weeks of the pandemic found that most countries suffered from insufficient involvement of relevant stakeholders [23]. Our interpretation is that the formal and legal structures that empower different actors in crisis management and CDC work in Norway turned out to be helpful in exposing differences in perspectives at the local and national level, and therefore contributed to arrival at agreed solutions.

Christensen et al. state that hybrid arrangements combining formal hierarchy and informal networks might be a promising compromise in crisis management [24]. In our study, the informants described how the formal hierarchical structures between national authorities, the County Governor, the municipalities, the MCM teams and the CMOs functioned as flexible frameworks, tolerating disagreements and the formation of informal networks between and within the levels.

### Trust and local authority

Trust is one of the foundations for effective crisis management [25]. Norwegians have high trust in the government and public sector [12]. Two main elements in the successful Norwegian handling of the pandemic were probably the extensive local authority in combination with present medical knowledge locally, trust and respect between different stakeholders [7]. The local CMO arrangement in Norway, with medical doctors in these positions, is to our knowledge quite unique compared to most other countries. In Sweden and Denmark, for example, the lowest level of medical CDC responsibility is regional. In England, the responsibility for public health was moved from the National Health Service to a local-level system in 2013 and is now led by Directors of Public Health who are not necessarily medical doctors. They do not have the legal option to make local infection-control decisions that is given to the Norwegian CMOs. Apart from that, the King’s Fund report, ‘Directors of public health and the COVID-19 pandemic’, describes many pandemic experiences similar to our findings [26].

The CMOs played an important role during the pandemic both internally as part of the municipality administration and crisis management and externally to the population. This role was based on their vital medical knowledge and trust from the local politicians, the administration and the public. The CMOs in our study expressed how their position made them feel personally responsible for their population. None of the CMOs in our study stepped out of their legal boundaries or were given a formal leadership role during the pandemic. The framework was flexible enough to give the CMOs enough leeway to act efficiently.

In this study, we chose to regard the first weeks of the pandemic as a crisis [3]. Crisis inflicts chaos and needs order. Crisis management involves multiple organisations at multiple levels. This calls for a combination of governance capacity and legitimacy, as well as flexibility and adaption [3,24]. Our findings show how the MCM teams and CMOs created informal networks to tailor make their early handling of the pandemic in tune with both national measures and local needs and challenges. The formation of contact structures between local businesses and CMOs is an example of what Boin and Bynander describe as emergent organisations [3]. These local adjustments were done within the frames of the principles of crisis management and legal possibilities. The crisis management in a pandemic is regulated by both the CDC Act and the regulations of crisis management in general. These laws do not perfectly align, especially when it comes to the role of the County Governor. Our informants described how this caused confusion and disagreement, demonstrating the importance of having an adequate formal structure for crisis management, but also the national, regional and local governments’ ability to find solutions despite shortcomings of formal structure.

### Strengths and limitations

In this exploratory study, we interviewed a purposive sample of local CMOs and MCM team members representing different geographical regions, backgrounds and experiences. Some municipalities had experienced outbreaks of COVID-19; some had not. Some had decided local infection-control measures; some had not. All the MCM teams conveyed how they leaned heavily on the CMOs’ competence and judgements. A weakness with our sample is that we did not have participants from municipalities with unstable CMO coverage [27]. A locum CMO might have a less important role than the descriptions in our material. The combination of individual interviews and focus-group interviews gave rich insight into participants’ experiences. With our focus on rurality and local perspectives, we purposely looked for experiences that could shed light on these aspects. Researchers with another focus would have emphasised other perspectives.

## Conclusions

The strong municipal responsibility and the quite unique arrangement with local medical CMOs in every municipality with a legal right to decide temporary local infection-control measures seem to have facilitated a constructive balance between top-down and bottom-up decision making, also contributing to give rural municipalities’ situation national visibility and relevance. The tension between rural and national actors that arose due to local infection-control measures in the first weeks of the pandemic in Norway, and the following dialogue and mutual adjustment of perspectives, led to a fruitful balance between national and local measures in Norway’s handling of the COVID-19 pandemic.
